# Linkage and Haplotype Association of Cognitive SuperAging to Chromosome 14 in the Midwestern Amish

**DOI:** 10.1002/alz.090963

**Published:** 2025-01-03

**Authors:** Daniel A. Dorfsman, Michael B. Prough, Alex V. Gulyayev, Laura J. Caywood, Jason E. Clouse, Sharlene D. Herington, Susan H. Slifer, Larry D. Adams, Renee A. Laux, Yeunjoo E. Song, Audrey Lynn, Sarada L. Fuzzell, Sherri D. Hochstetler, Kristy L. Miskimen, Leighanne R. Main, Ping Wang, Yining Liu, Noel C. Moore, Paula K. Ogrocki, Alan J. Lerner, Jeffery M. Vance, Michael L. Cuccaro, Margaret A Pericak‐Vance, William K. Scott

**Affiliations:** ^1^ Dr. John T. Macdonald Foundation Department of Human Genetics, University of Miami Miller School of Medicine, Miami, FL USA; ^2^ John P. Hussman Institute for Human Genomics, University of Miami Miller School of Medicine, Miami, FL USA; ^3^ Department of Population and Quantitative Health Sciences, Case Western Reserve University School of Medicine, Cleveland, OH USA; ^4^ Cleveland Institute for Computational Biology, Case Western Reserve University, Cleveland, OH USA; ^5^ Department of Genetics and Genome Sciences, Case Western Reserve University School of Medicine, Cleveland, OH USA; ^6^ Department of Nutrition, Case Western Reserve University School of Medicine, Cleveland, OH USA; ^7^ Department of Neurology, Case Western Reserve University School of Medicine, Cleveland, OH USA; ^8^ Department of Neurology, University Hospitals Cleveland Medical Center, Cleveland, OH USA; ^9^ Case Western Reserve University School of Medicine, Cleveland, OH USA

## Abstract

**Background:**

While Alzheimer’s disease and dementia prevalence increase with age, some older adults retain cognitive performance equal to those in mid‐life. One group, referred to as SuperAgers (SA), are ≥ 80 years old and demonstrate episodic memory function at or above the level expected for a middle‐aged adult. Genetic studies of SA may reveal heritable factors that promote superior cognition in older adults. This study utilizes an Amish sample to identify genetic factors underlying SA using both pedigree‐based and pedigree‐free analytical methods.

**Method:**

Adult Amish participants underwent a comprehensive neuropsychological examination. Those aged ≥ 80 with episodic memory ≥ the mean for ages 35‐44 and performance in non‐episodic memory tasks (e.g., language, executive function tests) within one standard deviation or better for their own age were considered SA (N = 83). Using family records, SA subjects were grouped into 16 pedigrees for parametric and non‐parametric linkage analysis in MERLIN. To assess the evidence of a shared haplotype across pedigrees, pairwise identical‐by‐descent segments were generated in the overall sample using iLASH and then assembled into multi‐sample IBD haplotype clusters using DASH. IBD haplotype frequencies were compared between SA and a comparison group comprised of age‐matched cognitively unimpaired non‐SA, as well as individuals exhibiting cognitive impairment (N = 304). Genetic variants within haplotypes of interest were tested for association with SA in GENESIS.

**Result:**

In the parametric analysis, HLOD scores > 3 were detected on chromosomes 1 (peak at 44 Mb), 2 (203 Mb), 7 (30 Mb), 14 (100 Mb), and 20 (17 Mb). IBD haplotypes within a 5Mb region centered around the peak linkage signals were further assessed. The strongest haplotype‐based association was observed on a ∼660 Kb segment on chromosome 14 (α_Bonferroni‐adjusted_ = 1.4e‐4, p = 3.16e‐5). Single‐variant association testing within the boundaries of the chromosome 14 haplotype identified 6 strongly correlated intronic/intergenic variants enriched in SA (MAF = 0.01, α_Bonferroni‐adjusted_ = 1.8e‐5, p = 5.22e‐8).

**Conclusion:**

This investigation found evidence of linkage and association of SA to a region on chromosome 14. Multiple genes in the region including *DLK1*, implicated in hippocampal neurogenesis, represent candidate targets for further study.

